# p63 transcriptionally regulates the expression of matrix metallopeptidase 13

**DOI:** 10.18632/oncotarget.1778

**Published:** 2014-02-14

**Authors:** Ivana Celardo, Alexey Antonov, Ivano Amelio, Margherita Annicchiarico-Petruzzelli, Gerry Melino

**Affiliations:** ^1^ Medical Research Council, Toxicology Unit, Leicester University, Leicester LE1 9HN, UK; ^2^ Saint-Petersburg State Institute of Technology, Saint- Petersburg, Russia; ^3^ Biochemistry Laboratory IDI-IRCC; Department of Experimental Medicine and Surgery; University of Rome “Tor Vergata”; Rome, Italy

**Keywords:** p63, MMP13, Collagenase, skin, metastasis

## Abstract

p63 is a transcriptional factor belonging to p53 family of genes. Beside the role in cancer, partially shared with p53 and the other member p73, p63 also plays exclusive roles in development and homeostasis of ectodermal/epidermal-related organs. Here we show that p63 transcriptionally controls the expression of the matrix metallopeptidase 13 (MMP13). p63 binds a p53-like responsive element in the human promoter of MMP13, thus promoting the activation of its transcription. The catalytic activity of MMP13 is required in high invasion capacity of metastatic cancer cells, however, although p63 and MMP13 expression correlates in cancer patients, their co-expression does not predict cancer patient survival. Our results demonstrate that p63 directly controls MMP13 expression.

## INTRODUCTION

p63 is a p53-related transcriptional factor, which plays roles in development and cancer. Different promoter regions control the expression of two N-terminal isoforms, TAp63 and ΔNp63. The P1 promoter, located upstream of exon 1, controls expression of a transactivation domain (TA)-containing isoform (TAp63), whereas the P2 promoter controls, located in intron 3, promotes the expression of the N-terminal truncated isoform (ΔNp63), which lacks the first TA domain. Alternative splicings at the 3' region of p63 pre-mRNA generates three additional C-terminal isoforms, p63α (full length), p63β (lacking exon 13) and p63γ (lacking exons 11-14).

In response to the DNA damage TAp63 can induce cell cycle arrest and apoptosis, partially overlapping function of the others p53 family members, p73[1-4] and p53 [5-10]. In addition, overlapping involvement of all three family members have also been described in metabolism of glutamine[11-15] and serine/glycine[16, 17]. TAp63 has been detected at low levels in different tissues, especially following stress[18]. However, the predominant expression is confined in oocytes, where, in response to genotoxic insults, promotes the induction of BH3-only pro-apoptotic BCL-2 family members, PUMA [19-23] and NOXA[24-30]. Thus, TAp63 has been named the ‘guardian of the female germline’ [31, 32]. However, TAp63 controls also other cell cycle regulators and pro-apoptotic genes, some of which shared with p53 and p73, including p21[33], Casp-1,2,3,4,5,8,9 [34-37], TNF, TNF-R1, TRAL-R1, -R2 [38-41]. TAp63 also plays a role in metastasis suppression, repressing TGF-β-mediated invasiveness. Mutant-p53 forms a ternary complex with Smad and p63 [42-46], antagonizing the p63 metastasis suppression function. Overall, the different N-terminal isoform functions, the tissue-specific expression and interaction with other paralogue genes p53 and p73 [47] arise high complexity to p63 function in cancer (for rev. see [48]).

ΔNp63 is master regulator of epithelial development and differentiation. p63^−/−^ (lacking all p63 isoforms) [49-52] and ΔNp63^−/−^ selective null-mice [53] present severe development abnormalities, including limb truncations, craniofacial malformations and the lack of an intact epidermis. Moreover, these animals are born without teeth, mammary glands, prostate or skin appendages – structures that are highly dependent on proper epithelial-mesenchymal interactions. ΔNp63 directly drives epidermal morphogenesis and preserves epidermal homeostasis, maintaining basal keratinocyte proliferative potential in adult normal epidermis [54, 55], through regulation of crucial transcriptional target genes[56-60] and microRNAs[61-63]. Recently, Flores group has revealed a role for ΔNp63 in the reprogram adult somatic cells into multipotent stem cells. They showed that ΔNp63^−/−^ epidermal cells express a subset of markers present in embryonic stem cells and fibroblasts induced to pluripotency using Yamanaka factors [64]. p63 has also been proven to be involved in cutaneous wound healing [65], however still little is known about p63 targets genes during re-epithelialization.

Human matrix Metallopeptidase 13 (MMP13), also known as collagenase-3, is a proteolytic enzyme, identified in 1994 by Freij and colleagues [66]. The substrate specificity of MMP13 indicated that this protease exhibits high proteolytic activity toward fibrillar type I, II, and III collagens [67]. It plays crucial roles in physiological processes such as fetal bone development [68], skeletal development [69, 70], articular cartilage and synovial membrane functionality [71]. MMP13 is low expressed in proliferating keratinocytes, but highly induced at the leading edge of skin wound [72, 73]. Many factors can control keratinocyte migration [65, 74, 75], but the limiting process is the attachment of keratinocytes to fibrillar type I collagen and subsequent degradation of the collagen by collagenolytic MMPs [76-78]. MMP13 overexpression indeed promotes keratinocyte migration, mediating degradation of the collagen and consequent cell detachment from the basal membrane. It has also been proved that MMP13 is involved in re-epithelialization in wound healing in mice [77]. However, other studies raised a debate on this issue, since MMP13^−/−^ mice failed to demonstrate significant difference or showed acceleration of wound closure and re-epithelialization [79]. MMPs are generally considered important targets for human diseases where these enzymes have been found overexpressed and/or where excessive collagen degradation is involved[80, 81]. Pathological states associated to MMP13 upregulation are different type of cancers[82], osteoarthritis[83, 84], and rheumatoid arthritis[85]. In human cancer upregulation of MMP13 contributes to metastasis onset [72]. The catalytic activity of MMP13 may play a role in the high invasion capacity of cancer cells. Degradation of basement membrane is thought to be an early and critical event influencing tumor invasion and metastasis. Thus, MMP13 represents a negative prognostic factor for different cancers [82, 86, 87]. Recently, our group has identified MMP13 as a direct transcriptional target of the p63 homologous, TAp73[88].

Here we describe MMP13 as target of p63. p63 can indeed transcriptionally control the expression of MMP13 by binding its promoter gene and promoting its expression. This finding indicates a novel target gene of p63 that can help to elucidate p63 role in cancer and in the re-epithelialization process during wound healing.

## RESULTS AND DISCUSSION

### p63 promotes MMP13 expression

In a gene expression-profiling analysis on TAp63 and ΔNp63 overexpressing cells performed by microarray approaches, we identified important subsets of genes, which potentially might be transcriptionally regulated by p63. Among the genes, whose expression was modulated by both TAp63 and ΔNp63 isoforms, we focused on MMP13.

**Figure 1:**
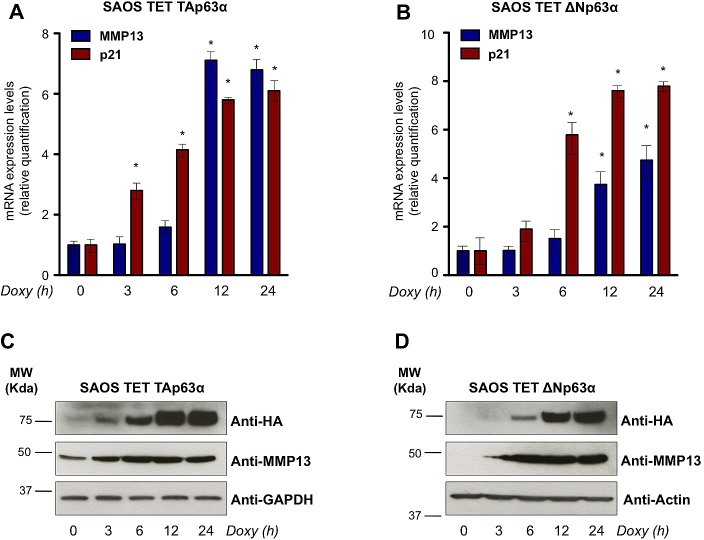
p63 upregulates mRNA and protein levels of MMP13 (a,b) mRNA expression of MMP13 was induced by both p63 isoforms in a time-dependent manner. Levels of MMP13 and of the previously described p63 target gene, p21, were evaluated by real time qPCR following the TAp63α (a) and ΔNp63α (b) induction after 3, 6, 12, 24 hours (h) of 4μg/ml doxycycline (Doxy) treatment in SaOs-2 inducible cells. Relative expression of MMP13 and p21 was normalized using the housekeeping gene, TBP, and calculated as fold induction. Data represent mean ± SD of three different experiments. *, p < 0.01 (c,d) MMP13 protein induced by TAp63α (c) and ΔNp63α (d) at the same time indicated for qPCR. Western blot analysis was performed using the indicated antibodies. GAPDH or Actin were evaluated as control. The experiment is representative of three independent experiments.

To validate the microarray data, we employed SaOs-2 Tet On cell lines, where expression of TAp63α or ΔNp63α can be induced by the tetracycline analog, doxycycline. Real time qPCR, performed in a time course analysis from 0h to 24h of doxycycline treatment, showed a significant induction of exogenous TAp63α and ΔNp63α and a consistent upregulation of the previously reported p63 transcriptional target, p21 (Fig. 1A,B). qPCR on the time course induction of both TAp63α and ΔNp63α SaOs-2 cell lines showed a time-dependent upregulation of MMP13, confirming the result of the microarray analysis (Fig. 1A,B). To confirm this regulation at protein level we performed western blot analysis on TAp63α and ΔNp63α SaOs-2 cell lines upon doxycycline treatment. Consistently, MMP13 protein levels were upregulated in time-dependent fashion, when HA-tagged TAp63α and ΔNp63α were induced (Fig. 1C,D). Notably, detectable levels of MMP13 were already observed in absence of p63 overexpression (time 0h). Being all p53-family members undetectable in parental SaOs-2 cell line, this observation suggested that alternative mechanisms are also involved in transcriptional regulation at least of basal level of MMP13. Overall, our data suggest that p63 controls expression of MMP13.

### p63 directly binds human MMP13 promoter

**Figure 2:**
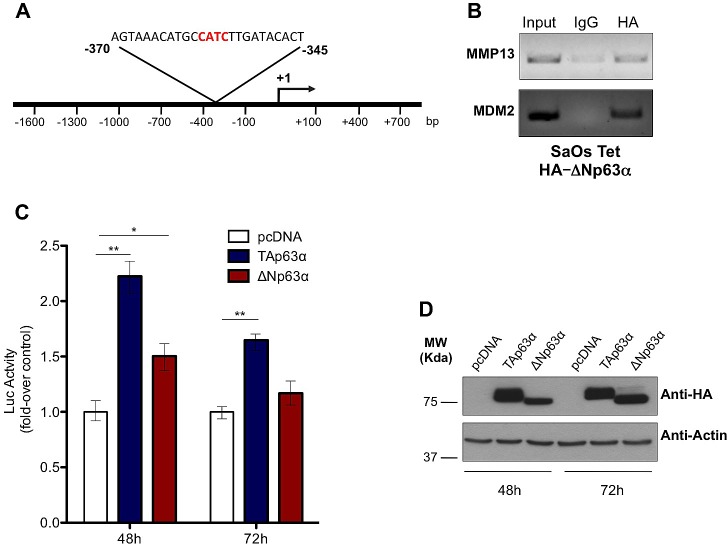
p63 directly binds and transactivates MMP13 promoter (a) Schematic map of human MMP13 promoter region with the p53-like RE. The insert shows the sequence of p53 RE, located between -345 and -370 bp upstream of transcription-start site. (b) SaOs-2 cells were induced with 4μg/ml doxycycline for 24h. The sonicated chromatin was bound to ΔNp63α-HA and amplified by PCR with MMP13 primer that recognizes the p53-response element. ChIP on MDM2 promoter was performed as a positive control. Mouse IgG antibody was used as negative control of the ChIP procedure. (c) Both TAp63α and ΔNp63α isoforms transactivate MMP13 promoter at 48h. The MMP13 promoter activity was evaluated after cotransfection with pcDNA vector, TAp63α ΔNp63αplasmids. The luciferase assay was performed after 48h and 72h of cotransfection in 293T cells and was normalized with Renilla luciferase vector. The graphs show a mean ± SD of three different experiments. **, p < 0.01; *, p < 0.05; (d) Western blot analysis on lysates from luciferase assay was performed as control of p63 protein overexpression. Actin was used as loading control.

To deeply investigate the mechanisms by which p63 controls MMP13 expression, we hypostasized that a direct binding of p63 on MMP13 gene promoter might be responsible of the transcriptional regulation of the gene. To screen MMP13 promoter sequence in order to identify p63 (p53-like) responsive elements (RE), we employed MatInspector Professional software [89]. We analyzed the first 1.6 Kb upstream the transcriptional start, searching for the p53-like consensus motifs (matrix V$P53F/P5301), which contained the core sequence CATC. The in silico analysis suggested that region between -345/-370 contained a putative consensus sequence, which potentially might be bound by p63 (Fig. 2A). To experimentally prove the direct binding of p63 on this consensus sequences we designed a ChIP assay. To perform ChIP we used ΔNp63 SaOs-2 Tet On cells, where expression of ΔNp63 was induced for 24h by doxycycline. The immunoprecipitated chromatin was then subjected to a PCR designed to amplify the p53-RE region included in the MMP13 promoter (Fig. 2A). PCR band was specifically detected only in the anti-HA immunoprecipitated chromatin, when compared with anti-IgG immunoprecipitated control chromatin. Notably, as p63 binding on MDM2 promoter region was already reported, PCR to detect p53 responsive element in MDM2 promoter region was performed as positive control of the experimental procedure. Hence, our ChIP analysis demonstrated the physical interaction of p63 with the p53-like RE in the MMP13 promoter, strongly suggesting that MMP13 might be a *de novo* transcriptional target gene of p63 (Fig. 2B).

### p63 activates transcriptional regulation of MMP13 promoter

To elucidate whether the direct binding of p63 on MMP13 promoter was functional, the -1600 bp upstream the transcriptional start were inserted into luciferase reporter plasmid to evaluate the transcriptional ability of p63 on this genomic region (Fig. 2A). The promoter was then cotransfected into 293T cells together with HA-tagged TAp63α or ΔNp63α or empty control vector. 48h after transfection, increased luciferase activity confirmed a significant transcriptional activation of MMP13 promoter by both TAp63α and ΔNp63α (respectively 2.3- and 1.5-fold increase). Consistently, after 72h of transfection similar trend was confirmed, although only TAp63α overexpressing cells provided a statistical significant increase (Fig. 2C). Notably, western blot analysis confirmed good transfection efficiency for both TAp63α and ΔNp63α at the two time points (Fig. 2D). These data demonstrate that p63 binding on the binding sequences located at -370/-345 from transcription-start site is functionally active to promote MMP13 expression. However, the qPCR results shown in figure 1, where p63-dependent upregulation of MMP13 showed up to 7-fold increase, would suggest that, besides this p53-RE, additional consensus sequences might be involved in the transcriptional regulation of MMP13 by p63.

### p63 correlates with MMP13 expression in cancer patients

**Figure 3:**
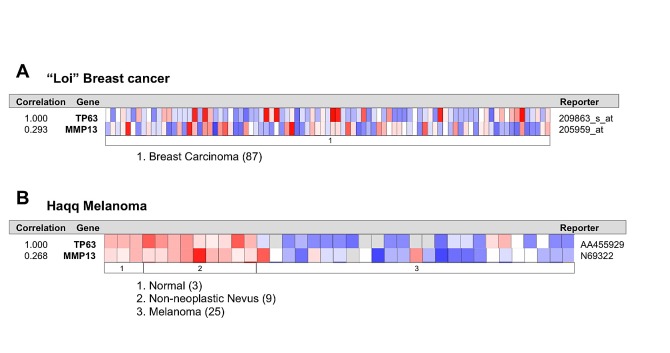
p63 and MMP13 expression directly correlates in human breast and melanoma cancers (a,b) Expression data from Oncomine website (www.oncomine.org). We used the following filters: gene ‘TP63’ Analysis Type: ‘Coexpression analysis’ Cancer Type: Breast (a) or Melanoma Cancer (b). The colour changes according to a weaker (blue) or higher (red) expression, passing by white, with fluctuating colour intensity. The reporters indicate probes used for the analysis.

**Figure 4:**
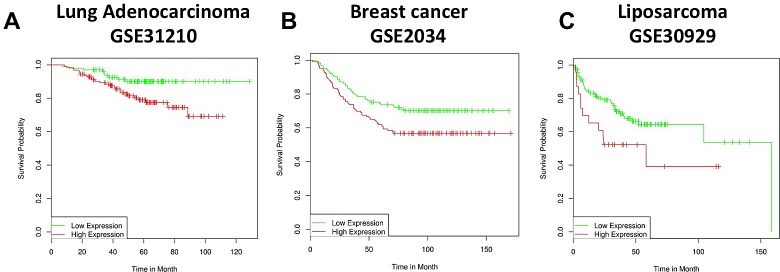
MMP13 expression is a negative prognostic marker in human cancers (a-c) Three different human cancer datasets have been analysed: GSE31210 (Lung adenocarcinoma a), GSE2034 (Breast cancer b), GSE30929 (Liposarcoma c). The panels represent patient survival estimation of MMP13 high expressing compared to low expressing groups.

**Figure 5:**
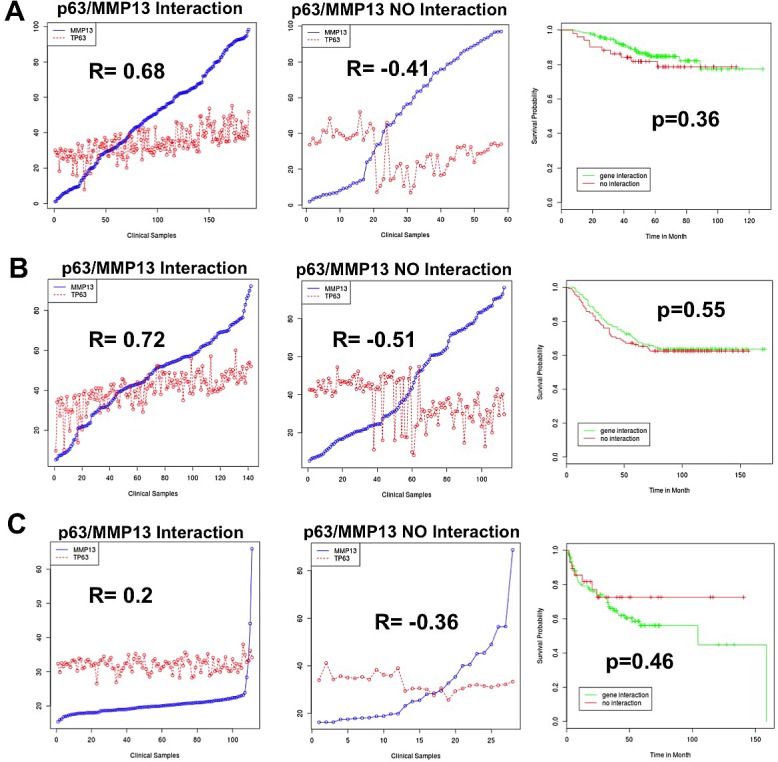
p63/MMP13 correlation does not function as prognostic marker in cancer Three different human cancer datasets have been analysed: lung adenocarcinoma GSE31210 (a), breast cancer GSE2034 (b), liposarcoma GSE30929 (c). (a-c) Left and central panels respectively represent p63 and MMP13 expression profiles in the cohorts 1 (p63/MMP13 Interaction) and cohorts 2 (p63/MMP13 NO Interaction). Right panels represent patient survival estimation in the two groups.

To confirm the existence and to assess the relevance of p63/MMP13 axis, we searched for expression correlation of these two genes in human cancer patient datasets. Breast cancer represents a heterogeneous group of diseases with different biological and morphological features, although strong efforts have been placed in identification of biomarkers of high-grade/metastatic forms of this disease[90-92], still much is expected to improve prognostic and predictive marker assessment. Melanoma is among the most aggressive cancer, with high incidence of metastasis and advanced stages progression [93-96]. Therefore to confirm p63/MMP13 relevance in cancer, we selected two cancer datasets: a breast carcinoma cohort [97] of 87 samples and a melanoma cohort, which included 25 melanomas [98], 9 non-neoplastic nevus and 3 normal samples. Coexpression analysis showed direct association between p63 and MMP13 mRNA level in both the datasets analysed. The breast cancer dataset presented a correlation factor of 0.293, while the melanoma dataset of 0.298 (Fig. 3A,B). These data confirm a coexpression and a direct correlation of p63 and MMP13 in human cancers. The melanoma dataset included normal and non-neoplastic specimens. Interestingly, a clear switch from high p63/MMP13 expression to low p63/MMP13 occurred in the transition towards the malignant state of melanocytes (Fig. 3B). To further investigate the function of p63/MMP13 in cancer, we decided to analysed MMP13 ability to function as prognostic factor of human cancer. We used three different publicly available datasets of human cancer, which included patient survival information (lung adenocarcinoma GSE31210, breast cancer GSE2034, and liposarcoma GSE30929). Patient datasets were clustered according to the expression of MMP13 (high expressing, low expressing). Kaplan-Mayer curve for survival estimation showed that a high MMP13 expression was predictive of poor survival (Fig. 4A-C), suggesting that MMP13 plays an important role in cancer aggressiveness. In order to elucidate whether p63 functions as a controller of MMP13 expression in cancer, we evaluated the biological consequence of p63/MMP13 coexpression in cancer patients. We selected the datasets analyzed in figure 4 (lung adenocarcinoma GSE31210, breast cancer GSE2034, and liposarcoma GSE30929) and we split the samples in two cohorts. The first cohort included all the samples to maximize positive correlation between p63 and MMP13, while in the second cohort all the other samples were included (Fig. 5). Thus, we clustered the dataset in two biological groups: one where p63/MMP13 axis was present (p63/MMP13 interaction), and second in which it was absent (p63/MMP13 NO interaction). Performing Kaplan-Mayer survival estimation the two cohorts did not show any significant difference in the survival expectation (Fig. 5). These data suggest that, although p63 is able to control MMP13 expression, it is very unlikely this axis plays any relevant role in cancer biology.

## CONCLUSION

p63 plays fundamental roles in development and homeostasis of the epidermis as well as in cancer. Here we have identified the matrix metallopeptidase 13, MMP13, as direct transcriptional target of p63. A wide overlapping involvement of all p53-family occurs in the transcriptional regulation of MMP13. We have indeed recently demonstrated that MMP-13 is a direct downstream target of p73 [88]. In addition wt p53 and mutant p53 (mp53) exert opposite regulation on MMP13 expression. Wt p53 represses MMP13, while mp53 promotes MMP13 expression in wt p53 expressing cells[99]. This regulation is in line with pro-metastatic ability of mp53 (gain-of-function feature), however our data do not suggest a direct involvement of p63/MMP13 axis in cancer progression. Our clinical analyses suggest that p63-dependent regulation of MMP13 has not direct impact on survival outcome of cancer patient. This observation tempts to speculate that MMP13 regulation by p63 may represent an important axis in epidermal wound healing and skin homeostasis. Being both available p63 and MMP13 animal models, further investigations will clarify the relationship between these two genes in order to provide a deeper understanding of their the biological relevance of their interaction.

## MATERIALS AND METHODS

### Cell cultures and Transfection

All cell lines were grown at 37°C in a humidified atmosphere of 5% CO_2_ in air. SaOs-2–Tet–On cells were maintained in RPMI, 10% (vol/vol) Tet-free FCS (Clontech), penicillin/streptomycin 1 unit/ml (Gibco) and 250mM L-glutamine (Gibco). A Tet-responsive transcriptional activator rtTA was used to obtain SaOs-2 clones with the inducible expression of TAα and ΔNα p63 isoforms (Tet–On system). 293T cells were maintained in RPMI, 10% (vol/vol) FCS (Invitrogen) 250 mM sodium pyruvate (Gibco), penicillin/streptomycin 1 unit/ml (Gibco) and 250 mM L-glutamine (Gibco).

### Cell transfection and luciferase assay

293T cells were seeded 24h before the transfection at density of 1 x 10^5^ in 12-well dishes. The day after, cells were co-transfected with 300ng/well of HA-tagged pGL3 vector or TAp63α and ΔNp63α plasmids, 100ng/well MMP13 luciferase reporter plasmid and 2ng/well pRL-cytomegalovirus vector. Transfection was carried out by Effectene reagent (Qiagen) according to the manufacturer's instructions. After 48h and 72h from the transfection, the luciferase activities of cellular extracts were detected using a Dual-Luciferase Reporter Assay System (Genecopoeia). Renilla luciferase activity was used to normalize the transfection efficiency. Light emission was measured over 5s using a luminometer (with Glomax 96 Microplate Luminometer, Promega (Madison, WI, USA)).

### Western Blotting

Proteins were extracted with RIPA buffer containing mixture inhibitors (Roche), followed by homogenization by using QIAshredder (Qiagen) and evaluation of concentration by using a Bradford dye-based assay (Bio-Rad). 50μg of total proteins were loaded on 10% SDS/PAGE and transferred to polyvinylidene difluoride membranes (Hybond; GE Healthcare), followed by immunoblotting with appropriate antibodies. The blots were then incubated with peroxidase linked secondary antibodies followed by enhanced-chemiluminescent detection using ECL kit (Amersham). Primary antibodies were used as followed: anti-HA 1 : 100 (Covance); anti-MMP13 1:500 (Millipore); anti-actin and anti-GAPDH 1 : 10.000 (Sigma-Aldrich).

### RNA extraction and quantitative real-time PCR

After 4μg/ml doxycycline induction, total RNA was extracted from TAp63α and ΔNp63α SaOs-2 cells by using the RNeasy Mini Kit (Qiagen) and quantified by spectrophotometric analysis. Reverse transcription from 1μg of total RNA was performed using InPromII kit (Promega); 1/5 of cDNA obtained was used for real-time PCR. The primers used for the real-time PCR were: MMP13 +ATATGACTATGCGTGGCTGGAAC, _CCATGTGTCCCATTTGTGGTGTG; p21 +TGGGGATGTCCGTCAGAAC, _GGCGTTTGGAGTGGTAGAAATC; TBP +TCAAACCCAGAATTGTTCTCCTTAT, _CCTGAATCCCTTTAGAATAGGGTAGA. SYBR green ready mix (Applied Biosystems) was used to perform the real-time PCR. For the quantity normalization we used TBP as housekeeping gene; relative quantification of gene expression was analyzed according to the method of 2 ^−ΔΔCt^ reported in the User Bulletin no. 2 and the Relative Quantification software version 1.3 of Applied Biosystems.

### Analysis of promoter region

Analysis of promoter region was performed using Math-Inspector Professional software and the TRANSFAC database on a region of 1600kb upstream of the transcription-start site of the human MMP13. The analysis highlighted a 25bp region containing a p53-like RE site. The MMP13 promoter plasmid was purchased from Genecopoeia (vector: pEZX-PG02).

### Chromatin immunoprecipitation assay

After induction of ΔNp63α expression obtained by doxycycline 24h treatment (4μg/ml), SaOs-2 cells were collected and fixed in 37% formaldehyde. Sonication was used to shear the chromatin, followed by immunoprecipitation with or without 10μl of anti-HA antibody (Covance) for 2h using ChIP assay kit (Invitrogen). For the PCR we used the following primers: MMP13 +GCACCTCCAAGTCATCAAGC, _TGTGGGAAGAAGCAGAGAGTAG; MDM2 + GGTTGACTCAGCTTTTCCTCTTG, _ GGAAAATGCATGGTTTAAATAGCC.

### Bioinformatics analyses

Gene expression datasets GSE31210, GSE2034, GSE30929 were downloaded from the GEO omnibus repository. Gene expression rank reflects relative mRNA expression level and is more consistent as it requires no normalization and thus introduces no normalization bias. Gene expression values were transformed into rank expression values on the scale from 100 to 0. Rank value 55 means 55 per cent of probes in the sample have lower measured expression values. We use Pearson coefficient as a measure of correlation between expression profiles. Our primary aim is to divide samples in the dataset in two cohorts so that to maximize positive correlation between expression profiles of p63 and MMP13 in cohort 1 and to minimize positive correlation in cohort 2. We start with all samples in cohort 1 and no samples in cohort 2. We compute correlation between p63 and MMP13 expression profiles in cohort 1 as well as compute the changes in correlation if one sample from the cohort would be removed. The sample with maximal effect on correlation (maximal increase in positive correlation) is moved from cohort 1 to cohort 2. The same procedure is repeated until there would be no one sample which could be removed from cohort 1, so to increase positive correlation between p63 and MMP13. The separation of patients into “cohort 1” and “cohort 2” along with survival information is then used to find any statistical differences in survival outcome. The R statistical package is used to perform survival analyses and to draw KMplots.
